# Numerical investigation of two-microbubble collapse and cell deformation in an ultrasonic field

**DOI:** 10.1016/j.ultsonch.2022.106252

**Published:** 2022-12-06

**Authors:** Seongjin Hong, Gihun Son

**Affiliations:** Department of Mechanical Engineering, Sogang University, 35 Baekbeom-ro, Mapo-gu, Seoul 04107, South Korea

**Keywords:** Ultrasound, Bubble collapse, Bubble-bubble interaction, Liquid jet, Cell deformation

## Abstract

Two-microbubble collapse near a spherical cell in an ultrasonic field is numerically analyzed by extending a level-set method for compressible multiphase flows with bubble and cell multiple interfaces. Computations performed with different bubble-bubble distances and size ratios demonstrate various bubble-bubble interactions, such as bubble coalescence, bubble repulsion and attraction, jet penetration into the bubble, and jet collision. The interactions between collapsing bubbles are found to produce strong liquid jet formation and result in significant cell deformation compared to single-bubble collapse. The optimal bubble-bubble distance and size ratio for cell deformation are presented via contour maps based on extensive computations. The influences of the ultrasonic amplitude and frequency on cell deformation are further investigated.

## Highlights

• A numerical investigation of two-microbubble interaction near a deformable cell in an ultrasonic field is presented.
• The interactions between the two collapsing bubbles produce a strong liquid jet and cause significant cell deformation compared to single-bubble collapse.
• A variety of bubble interactions and associated cell deformation are demonstrated depending on the bubble-bubble distances and size ratios.
• The optimal bubble-bubble distance and size ratio for cell deformation are presented via contour maps for various acoustic pressure amplitudes and frequencies.

## Nomenclature

B: parameter for Tait equation (Pa)
$D_{c}$: deformation from the initial cell surface (m)
f: acoustic frequency (Hz)
$L_{\mathrm{bb}}$: center-to-center distance between two bubbles (m)
$L_{\mathrm{cb}}$: center-to-center distance between cell and adjacent bubble (m)
n: unit normal vector
p: pressure (Pa)
$p_{A}$: acoustic pressure amplitude (Pa)
r,y: cylindrical coordinate (m)
R: radius (m)
t: time (s)
u: flow velocity vector (m/s)

**Greek symbols**
γ: parameter for the Tait and ideal gas equations
μ: viscosity (Pas)
σ: surface tension (N/m)
ρ: density ($\mathrm{kg}/m^{3}$)
τ: viscous stress (Pa)
ϕ: signed distance from the interface (m)

**Subscripts**
b: bubble
c: cell
f: fluid
o: initial
w: water
∞: ambient

## 1 Introduction

Microbubbles expanded by ultrasound in a liquid collapse violently, generating strong shock waves and liquid jets. The liquid jets are non-linear phenomena that occur in asymmetric bubble collapse due to the influence of walls, shock waves, or other bubbles [1]. The bubble collapse has been an important topic in a variety of environmental and medical applications, such as water treatment, surface cleaning, drug delivery, and cell perforation, as reviewed in Refs. [2], [3]. Fundamental studies have also been reported on ultrasound-driven droplet vaporization [4], [5], acoustic cavitation with non-condensable bubble nuclei [6], and bubble oscillation on a rigid boundary [7], [8], [9].

The interactions between multiple bubbles occurring in actual ultrasonic applications have been investigated experimentally, theoretically and numerically. Bremond et al. [10] conducted numerical and experimental studies on the expansion and collapse of similar-sized two and more bubbles on a solid surface with microcavities. They observed that the solid surface acts as a mirror, and analyzed the influence of bubble–bubble distance on bubble flattening, coalescence and liquid jet towards each other. Ochiai and Ishimoto [11] investigated repulsive or attractive behavior of two microbubbles using a compressible homogeneous model. They observed that the acoustic force between two bubbles is more complicated in the nonspherical condition, and characterized the two-bubble motions into repulsive, merging, periodically stable and breakup motions according to the initial bubble–bubble distance and size ratio. Huang et al. [12] studied the influence of acoustic amplitude on the interactions between microbubbles using a boundary element method. The translational behavior of two bubbles prevailed at low acoustic amplitudes below 0.5 atm, whereas non-spherical bubble oscillations with two opposing liquid jets occurred at high acoustic amplitudes. Recently, Shen et al. [13] studied the bubble interaction for the radial pulsations of microbubbles using a theoretical model. The pressure radiated from oscillating microbubbles was observed to suppress or enlarge the expansion of other bubble depending on the ultrasound frequency, the bubble–bubble distances and the number of bubbles. Wang et al. [14] conducted an analytical study of the interaction of two microbubbles focusing on bubble translation and showed that high acoustic frequencies and pressure amplitudes produce fast translational velocity, whereas their effects decrease for bubbles significantly larger than the resonance radius.

Mutual bubble collapse and resulting liquid jets have also been investigated for bubbles generated by spark discharge or laser irradiation. Chew et al. [15] performed an experimental study for spark-generated two bubbles and showed that two collapsing bubbles with a time difference tend to produce liquid jets away from each other. Similar experimental and numerical works [16], [17] were conducted for spark-generated or laser-induced two bubbles. They observed that strong interaction occurs when the bubble–bubble distance is less than the sum of the maximally expanded radii of individual bubbles, and anti-phase collapse of bubbles can change the jet direction and enhance the jet velocity.

The interaction between bubble collapse and nearby tissue or cell deformation, which is a very important issue in practical environmental and medical applications, has been investigated in several numerical studies. Freund et al. [18] studied the influence of shock wave-induced microbubble collapse on the deformation of a tissue treating the tissue as a viscous liquid. They indicated that the viscosity of the tissue has a significant influence on the penetration into the tissue of the liquid jet caused by bubble collapse. Kobayashi et al. [19] assumed a tissue as a compressible liquid and investigated the influence of the acoustic impedance of the tissue on bubble collapse and the nearby tissue deformation. Guo et al. [20] numerically analyzed the acoustically driven microbubble collapse near a red blood cell modeled as a compressible liquid. They found that cell deformation is enhanced by either reducing the acoustic frequency or increasing the initial size of the bubble. Recently, Zevnik and Dular [21], [22] analyzed the bubble-liposome and bubble-bacteria interactions by employing a fluid–structure hybrid computational method and demonstrated the mechanical behavior of the bio-structural deformation induced by bubble collapse in a high-pressure field. The microbubble around the cell showed spherical collapse or liquid jet away from the cell, depending on the bubble-cell size ratio and bubble-cell distance. The main cause of cell deformation was observed to be a strong shock wave generated by bubble collapse. However, few studies have been extended to the analysis of cell or tissue deformation under the multi-bubble conditions encountered in practical ultrasound applications, except for the experimental works of Forbes et al. [23] and Tomita et al. [24].

In this work, we numerically investigate two-microbubble collapse near a deformable cell in an ultrasonic field by extending a level-set (LS) method for compressible multiphase flows with bubble and cell multiple interfaces. The formation of liquid jets due to multi-bubble collapse and their influence on cell deformation are analyzed for various bubble–bubble distances and size ratios. The optimal parameters for liquid jet formation and cell deformation are presented through contour maps based on extensive computations. The influences of the ultrasonic pressure amplitude and frequency are further investigated.

## 2 Numerical analysis

The LS method used in our previous studies [25], [26], [27] of compressible multiphase flows is extended to investigate two-microbubble collapse near a deformable cell immersed in water under ultrasonic pulse conditions at 1 atm and 293 K. The cell is treated as a compressible liquid droplet as in the previous studies for the interaction of a single bubble with tissues [18], [19] and cells [28], [20].

**Fig. 1:**
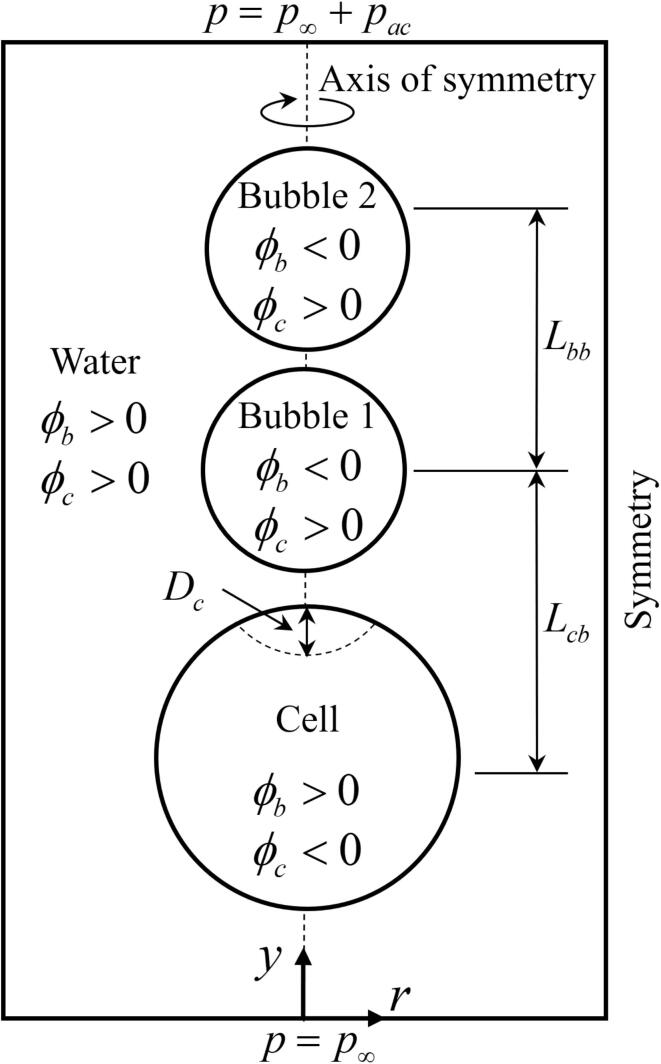
Schematic for analysis of bubble–bubble interaction near a deformable cell in an ultrasonic field.

In this work, we focus on the interaction of vertically arranged two microbubbles, referring to Fig. 1, the lower bubble closer to the cell is called as bubble 1, and the upper is called bubble 2. Computations are performed assuming that the microbubble motion and flow are axisymmetric. This assumption may not be so restrictive while the microbubble motion nearly axisymmetric due to the strong effect of strong surface. However, cavitation dynamic characteristics are generally three dimensional, and their numerical simulations are very challenging and time consuming. Such three-dimensional characteristics will be investigated in the future. The influences of surface tension, viscosity and compressibility are included using the typical properties of air, water and red blood cell [29], [30], while the effects of evaporation, condensation and heat transfer are not considered. Assuming that the flow is adiabatic, the following Tait Eq. (1) is used for cell and water phases, whereas the ideal gas Eq. (2) is used for the bubble phase.

(1) $$p_{c/w}=B_{c/w}[{\left(\frac{\rho_{c/w}}{\rho_{c/w,\infty }}\right)}^{\gamma_{c/w}}-1]+p_{\infty }$$

(2) $$p_{b}=p_{\infty }{\left(\frac{\rho_{b}}{\rho_{b,\infty }}\right)}^{\gamma_{b}}$$

Here, $p_{\infty }$ denotes atmospheric pressure and $\gamma_{b}=1.4$ is used for a bubble unless specified otherwise. The coefficients of Tait Eq. (1) for a cell are assumed to be equal to those of water, i.e., $B_{c/w}=331\;\text{MPa}$ and $\gamma_{c/w}=7.15$, considering that the water volume fraction in the cell reaches 80% [20], and typical bulk modulus and speed of sound of the biomaterials are similar to those of water. This approach using the equation of state for biomaterials has been applied in previous and recent numerical studies for the bubble-tissue and bubble-cell interactions [18], [19], [20], [28], [31], [32].

### 2.1 Governing equations

The conservation equations of mass and momentum are expressed as

(3) $$\frac{\partial \rho_{f}}{\partial t}+\nabla \cdot (\rho_{f}u_{f})=0$$

(4) $$\frac{\partial \rho_{f}u_{f}}{\partial t}+\nabla \cdot (u_{f}\rho_{f}u_{f})=-\nabla p_{f}+\nabla \cdot \tau_{f}$$

where

(5) $$\tau_{f}=\mu_{f}{\left[\nabla u+{\left(\nabla u\right)}^{T}-\frac{2}{3}(\nabla \cdot u)I\right]}_{f}$$

Here, the subscript *f* represents bubble ($\phi_{b}<0$), cell ($\phi_{c}<0$) and water ($\phi_{b}>0\cap \phi_{c}>0$) phases, respectively, as depicted in Fig. 1.

### 2.2 Interface conditions

At the bubble-water interface ($\phi_{b}=0$) and the cell-water interface ($\phi_{c}=0$), the matching conditions are expressed as follows:

(6) $$u_{b/c}-u_{w}=0$$

(7) $$[(p_{b/c}-p_{w})I+\tau_{w}-\tau_{b/c}]\cdot n_{b/c}=\sigma_{\mathrm{bw}/\mathrm{cw}}\kappa_{b/c}n_{b/c}$$

where

(8) $$n_{b/c}=\nabla \phi_{b/c}/|\nabla \phi_{b/c}|$$

(9) $$\kappa_{b/c}=\nabla \cdot n_{b/c}$$

The LS functions $\phi_{b}$ and $\phi_{c}$ are updated by the following advection and reinitialization equations,

(10) $$\frac{\partial \phi_{b/c}}{\partial t}+u\cdot \nabla \phi_{b/c}=0$$

(11) $$\frac{\partial \phi_{b/c}}{\partial t^{*}}+\frac{\phi_{b/c}}{\sqrt{\phi_{b/c}^{2}+h^{2}}}\left(1-|\nabla \phi_{b/c}|\right)=0\;\mathrm{for}\;\left|\phi_{b/c}\right|>\frac{h}{2}$$

where $t^{*}$ and *h* are an artificial time for iterative calculation and grid size, respectively, and the LS functions are not reinitialized in the near-interface regions of $|\phi_{b/c}|<h/2$ for better volume conservation [25], [26].

### 2.3 Discretization

The governing equations are discretized using the ghost fluid method to efficiently apply the interface conditions and the consistent transport method for multiphase flows with large density ratios. The semi-implicit pressure correction method [33] is applied to avoid severe time step limitation in compressible flows. Further details for temporal and spatial discretizations are described in the previous works [25], [26], [27].

### 2.4 Model validation

To validate the present numerical method, we consider a stationary air bubble of $R_{\mathrm{bo}}=2\;\mu \text{m}$ immersed in water, which is subjected to an ultrasonic wave [34]. To apply the same computational conditions with Ref. [34], viscous stresses are not included and an isothermal condition ($\gamma_{b}=1$) is used for $R_{b}⩾R_{\mathrm{bo}}$ and an isentropic condition ($\gamma_{b}=1.4$) for $R_{b}<R_{\mathrm{bo}}$. The initial bubble is at (r,y)=(0,0) in a cylindrical region of $r⩽250R_{\mathrm{bo}}$and $-750R_{\mathrm{bo}}⩽y⩽250R_{\mathrm{bo}}$. We use fine grids of $h=0.05R_{\mathrm{bo}}$ in a region close to the bubble ($r⩽5R_{\mathrm{bo}},|y|⩽5R_{\mathrm{bo}}$), whereas nonuniform grids in the other region. The pressure condition of $p_{\infty }+p_{\mathrm{ac}}$ is applied to $y=250R_{\mathrm{bo}}$, where the acoustic pressure $p_{\mathrm{ac}}$ is expressed as

(12) $$p_{\mathrm{ac}}=-p_{A}\sin(2\pi \mathrm{ft})\;\mathrm{if}\;t⩽N/f$$

(13) $$p_{\mathrm{ac}}=0\;\mathrm{if}\;t>N/f$$

**Fig. 2:**
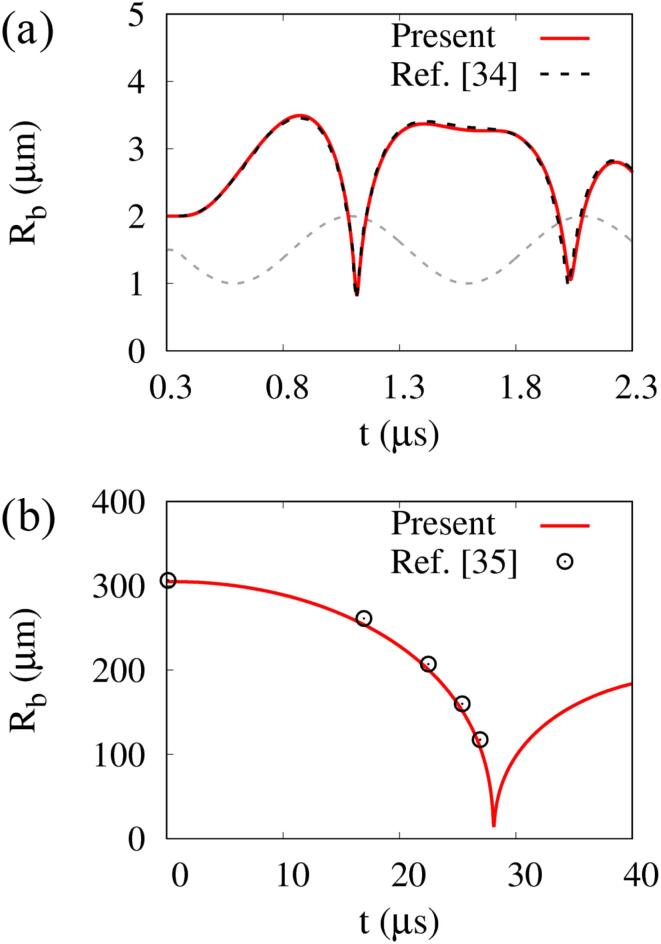
Validation test of the computed bubble radius change with: (a) previous numerical data [34] for a ultrasound-induced bubble oscillation, and (b) experimental data [35] for a laser-induced bubble oscillation. The gray dashed line represents the profile of water pressure at y=0 without a bubble.

In this validation test, we use $p_{A}=0.12\;\text{MPa},f=1\;\text{MHz}$ and N=2. The first calculation was conducted without including a bubble under an ultrasonic condition of $p_{\infty }+p_{\mathrm{ac}}$ at the upper boundary. The ultrasonic pressure wave travels with the speed of sound and arrives at the plane of y=0 near t=0.3μs. The temporal change of water pressure at y=0 has the same amplitude and frequency as $p_{A}=0.12\;\text{MPa}$ and f=1MHz. The next calculation result including a bubble at y=0 is plotted in Fig. 2a. Here, the gray dashed line represents the profile of water pressure at y=0 in the first calculation and the bubble radius is evaluated as $R_{b}={\left(3V_{b}/4\pi \right)}^{1/3}$ from the integrated bubble volume $V_{b}$. During the first negative pressure pulse period, the initial bubble grows to reach a maximum radius of $R_{b,\max}=1.75R_{\mathrm{bo}}$. As the pressure pulse turns positive, the bubble shrinks rapidly, and then oscillates during the next cycle of negative and positive pulses. The computed bubble radius change is in good agreement with the previous numerical result [34].

The present numerical method was also tested using the experimental data of Sankin et al. [35] for laser-induced microbubble oscillation in water. This test considers a microbubble of $R_{b,\max}=305\;\mu \text{m}$ and ambient water pressure of 1 atm as in Ref. [35], assuming that the bubble pressure at $R_{b,\max}$ is 2340 Pa. As depicted in Fig. 2b, the bubble shrinks due to the pressure difference with the ambient water and collapses near t=28μs. The computed bubble radius change agrees well with the previous experimental data [35].

### 2.5 Computational conditions

In the present computations of ultrasound-induced interactions of two microbubbles near a cell, as depicted in Fig. 1, we use the following air, water and red blood cell properties [29], [30]: $\rho_{b,\infty }=1.2\;\text{kg}/{\text{m}}^{3},\mu_{b}=1.9\times 10^{-5}\;\text{Pa}\;\text{s}$, $\rho_{w,\infty }=998\;\text{kg}/{\text{m}}^{3},\mu_{w}=1\times 10^{-3}\;\text{Pa}\;\text{s}$, $\rho_{c,\infty }=1110\;\text{kg}/{\text{m}}^{3},\mu_{c}=5\times 10^{-3}\;\text{Pa}\;\text{s}$, $\sigma_{\mathrm{bw}}=7.3\times 10^{-2}\;\text{N}/\text{m}$, and $\sigma_{\mathrm{cw}}=6.6\times 10^{-6}\;\text{N}/\text{m}$.

A spherical cell of $R_{\mathrm{co}}=10\;\mu \text{m}$ is initially located at (r,y)=(0,-10μm). The initial radius $R_{b2o}$ of bubble 2 and the initial center-to-center distance $L_{\mathrm{bbo}}$ between the bubbles vary while keeping the initial radius of bubble 1 as $R_{b1o}=2\;\mu \text{m}$ and the initial center-to-center distance between bubble 1 and the cell as $L_{\mathrm{cbo}}=16\;\mu \text{m}$. Referring to the literature [15], [16], [17], normalized distance ${\overset{∼}{L}}_{\mathrm{bbo}}$ and bubble size ratios ${\overset{∼}{R}}_{\mathrm{bb},\max}$ and ${\overset{∼}{R}}_{\mathrm{bbo}}$ defined as Eq. (14) are introduced to analyze the two-bubble motions in an ultrasonic field.

(14) $${\tilde{L}}_{\mathrm{bbo}}=\frac{L_{\mathrm{bbo}}}{R_{b1,\max}^{*}+R_{b2,\max}^{*}},\;{\tilde{R}}_{\mathrm{bb},\max}=\frac{R_{b2,\max}^{*}}{R_{b1,\max}^{*}},\;{\tilde{R}}_{\mathrm{bbo}}=\frac{R_{b2o}}{R_{b1o}}$$

Here, the maximum radii $R_{b1,\max}^{*}$ and $R_{b2,\max}^{*}$ of a single bubble are determined using the present numerical method or by solving the following Rayleigh-Plesset (RP) equation [6].

(15) $$\rho_{l}R_{b}{\overset{¨}{R}}_{b}=-\frac{3}{2}\rho_{l}{\overset{˙}{R}}_{b}^{2}+p_{b}-(p_{\infty }+p_{\mathrm{ac}})-\frac{2\sigma_{\mathrm{bw}}}{R_{b}}-4\mu_{l}\frac{{\overset{˙}{R}}_{b}}{R_{b}}$$

The computed maximum bubble radii for various initial radii are plotted in Fig. S1 in the Supplementary information noting that $R_{b1,\max}^{*}=5.71\;\mu \text{m}$ for $R_{b1o}=2\;\mu \text{m}$.

## 3 Results and discussion

The interaction between a single microbubble and a deformable cell is first computed for $R_{\mathrm{bo}}=2\;\mu \text{m}$ and $L_{\mathrm{cbo}}=16\;\mu \text{m}$ to investigate the effects of the direction of ultrasonic wave, bubble-cell distance, surface tension, viscosity and compressibility of the liquid on the bubble dynamics and cell deformation.

### 3.1 Ultrasound-induced oscillation of a single microbubble near a cell

For computations of interactions between a single microbubble and a deformable cell, we consider a large axisymmetric domain of $r⩽250R_{\mathrm{bo}}$ and $-750R_{\mathrm{bo}}⩽y⩽250R_{\mathrm{bo}}$. In the present computations, we use the symmetry boundary condition at $r=250R_{\mathrm{bo}}$ and the pressure boundary condition at $y=-750R_{\mathrm{bo}}$. The large domain is chosen to avoid the influences of non-physical reflections on bubble oscillations and cell deformations. We consider the region of uniform fine grids for $r⩽7.5R_{b1o}$ and $-12.5R_{b1o}⩽y⩽7.5R_{b1o}$ and the grid size of $h=R_{\mathrm{co}}/100$ is used for efficient computations based on the grid convergence test, which is plotted in Fig. S2 in the Supplementary information.

**Fig. 3:**
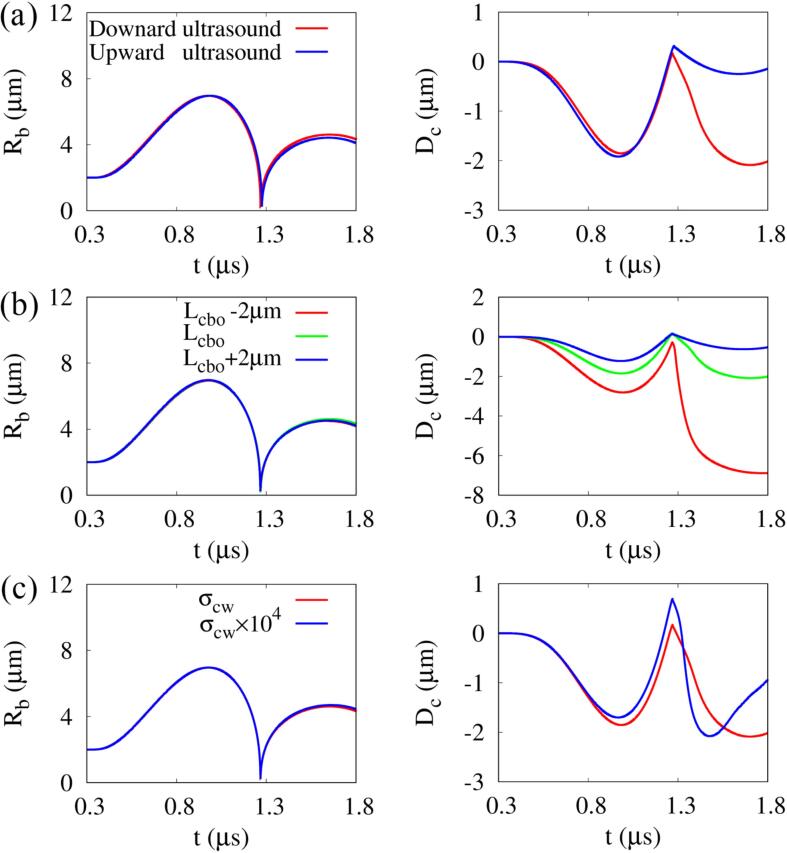
Temporal change of bubble radius (left) and cell deformation (right) associated with single bubble oscillation near a deformable cell at f=1MHz and $p_{A}=0.4\;\text{MPa}$ depending on: (a) ultrasonic wave direction, (b) bubble-cell distance, and (c) cell surface tension.

Fig. 3 shows the effects of ultrasound direction, bubble-cell distance and cell surface tension on the bubble growth and cell deformation at $f=1\;\text{MHz},p_{A}=0.4\;\text{MPa}$ and N=1. The calculation of the upward ultrasound can be performed by exchanging the y-direction boundary conditions. The change in bubble radius according to the ultrasound direction, bubble-cell distance, and cell surface tension shows little difference, and the maximum expansion radius and collapse time are nearly similar as $R_{b,\max}=6.96\;\mu \text{m}$ and t=1.27μs, respectively. However, the direction of ultrasound is observed to affect the direction of the liquid jet because it causes a different pressure gradients around the bubble. The liquid jet occurs in the direction of the pressure wave propagation, as indicated in Refs. [34], [36], which results in a decrease of the compressive cell deformation in the upward ultrasound case, as depicted in Fig. 3a. Regarding the liquid jet direction, some previous numerical studies reported that liquid jets can be directed away from the deformable boundaries [22], [28], [37]. However, the present study is different from the previous studies in that it considers the bubble oscillation caused by a traveling ultrasonic wave and the nearby cell deformation. In the present case, the direction of the liquid jet is determined by the wave propagation, as in Refs. [34], [36]. We focus on the downward ultrasound case, which is more effective for cell deformation.

In Fig. 3b, as the bubble-cell distance decreases and the repulsive and attractive interactions between the bubble and the cell become stronger, the liquid jet velocity and compressive cell deformation are pronounced, which is consistent with the previous observation of bubble-cell interaction in ultrasound [20]. The effect of cell surface tension is depicted in Fig. 3c. As the cell surface tension increases to $\sigma_{\mathrm{cw}}\times 10^{4}$, compressive cell deformation slightly decreases during bubble expansion and the surface-to-surface distance between the bubble and the cell decreases because the cell remains spherical. Due to the decrease in the bubble-cell distance, the expansive deformation of the cell increases during rapid bubble collapse, and the downward liquid jet velocity increases. This results in a steeper cell deformation after bubble collapse, however, the compressive cell deformation recovers faster compared to the base case. The shock pressure generated by bubble collapse is compared at (r,y)=(15μm,0) to avoid the influence of the liquid jet on the pressure. Although strong shock waves are generated during bubble collapse and re-expansion, the peak pressures show little difference depending on the bubble-cell distance and cell surface tension, as seen in Fig. S3. This is consistent with the experimental observation that the peak pressure of the shock wave is related to the maximum expansion size of the bubble [38].

**Fig. 4:**
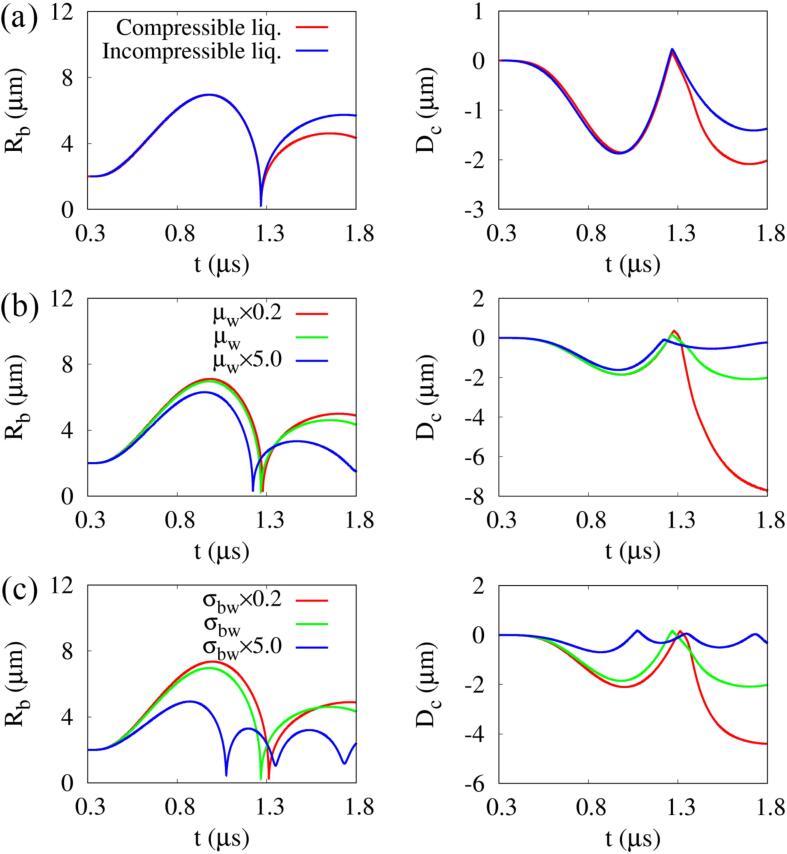
Temporal change of bubble radius (left) and cell deformation (right) associated with single bubble oscillation near a deformable cell at f=1MHz and $p_{A}=0.4\;\text{MPa}$ depending on: (a) liquid compressibility, (b) water viscosity and (c) bubble surface tension.

Fig. 4a shows the effect of liquid compressibility on the bubble growth and cell deformation. For an incompressible liquid case, the same ultrasonic pulse is applied at the bottom as well as top boundary and the result is plotted taking into account the propagation time of the pulse to compare with the compressible result. The liquid incompressibility appears to have little influence on the bubble growth and cell deformation during bubble expansion and contraction. However, the bubble re-expands larger than in the compressible liquid case because the loss of liquid kinetic energy, which is caused by the shock wave emission during bubble collapse and re-expansion, is not considered [5]. The incompressible liquid case also does not take into account the ultrasonic wave propagation, resulting in more spherical bubble collapse as well as reduced cell deformation. The effects of water viscosity and bubble surface tension on the bubble growth and cell deformation are depicted in Fig. 4b and c, respectively. As water viscosity increases, the maximum expansion radius of the bubble and the associated cell deformation decrease during bubble expansion. Thereafter, the compressive cell deformation by the liquid jet is also reduced because the liquid jet is weakened in the viscous water layer before reaching the cell surface. Similarly, an increase in the bubble surface tension is observed to decrease both the maximum expansion radius of the bubble and compressive cell deformation.

### 3.2 Ultrasound-induced oscillation of two microbubbles near a cell

For computations of bubble–bubble interactions near a deformable cell, the region of uniform fine grids is extended to $r⩽7.5R_{b1o}$ and $|y|⩽12.5R_{b1o}$ keeping the computational domain as described in the previous section. We choose $f=1\;\text{MHz},p_{A}=0.3\;\text{MPa}$ and N=1 as a base case. Computations are carried out for a wide range of $0.5⩽{\overset{∼}{L}}_{\mathrm{bbo}}⩽1$ and $0.7⩽{\overset{∼}{R}}_{\mathrm{bb},\max}⩽1$ ($0.5⩽{\overset{∼}{R}}_{\mathrm{bbo}}⩽1$). Since the bubble–bubble distance ($L_{\mathrm{bbo}}$) is less than 20μm, which is very short compared to the wave length of the ultrasound, the effect of the time difference of ultrasound reaching the two bubbles can be neglected.

**Fig. 5:**
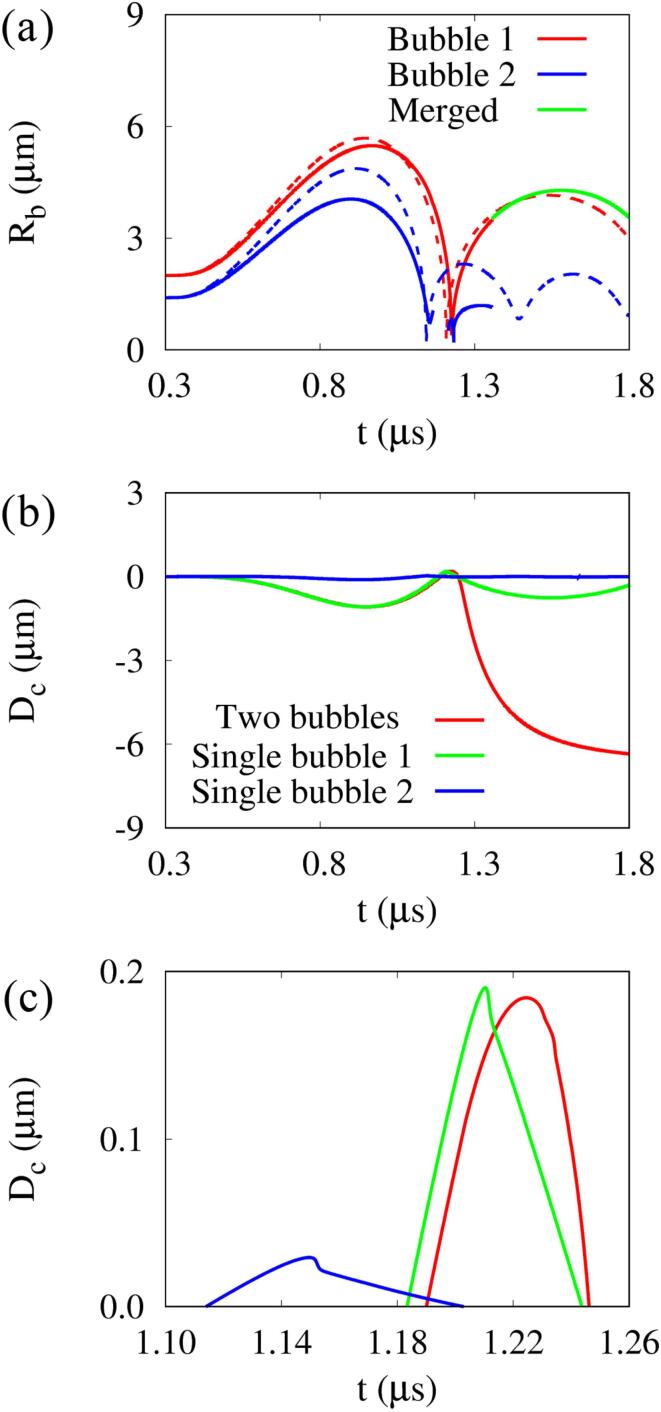
Temporal bubble and cell responses at $f=1\;\text{MHz},p_{A}=0.3\;\text{MPa},{\tilde{L}}_{\mathrm{bbo}}=0.8$ and ${\tilde{R}}_{\mathrm{bb},\max}=0.85$: (a) bubble radius, (b) and (c) cell deformation. The dashed lines represent the single-bubble cases with no opposing bubble.

Fig. 5 shows the computed bubble radius and cell deformation at ${\overset{∼}{L}}_{\mathrm{bbo}}=0.8$ and ${\overset{∼}{R}}_{\mathrm{bb},\max}=0.85$ (${\overset{∼}{R}}_{\mathrm{bbo}}=0.7$). The results for the two bubbles are compared to the single-bubble cases with no opposing bubble. The ultrasonic pulse starting with negative pressure arrives at y=0 near t=0.3μs. When the interaction between bubbles is not considered, the bubbles expand to $R_{b1,\max}=5.68\;\mu \text{m}$ and $R_{b2,\max}=4.87\;\mu \text{m}$, respectively, which are almost consistent with the results of $R_{b1,\max}^{*}=5.71\;\mu \text{m}$ and $R_{b2,\max}^{*}=4.88\;\mu \text{m}$ for a single bubble with no nearby cell (Fig. S1 in the Supplementary information). During the positive pressure pulse period, bubbles 1 and 2 begin to contract almost simultaneously at t=0.93μs. The bubble contraction proceeds faster than the bubble expansion by the positive acoustic pulse acting on the expanded bubble area. The small-sized bubble 2 collapses at t=1.14μs earlier than the collapse time t=1.21μs of bubble 1, and then the bubbles repeat expansion and contraction. On the other hand, considering the interaction between two bubbles, the expansion of bubbles decreases to $R_{b1,\max}=5.48\;\mu \text{m}$ and $R_{b2,\max}=4.05\;\mu \text{m}$, respectively. It is noted that the smaller bubble is more influenced by the larger. This can be explained by the theoretical equation $p=(\rho_{l}/r)d(R_{b}^{2}{\overset{˙}{R}}_{b})/\mathrm{dt}$ for the pressure around an oscillating bubble [13], which indicates that the larger the bubble size and the faster the expansion rate, the greater the influence on the surroundings. The expanding flows around the two bubbles repel each other so that the bubbles do not merge even under the condition of ${\overset{∼}{L}}_{\mathrm{bbo}}<1$. This was also observed in the two-bubble expansion and collapse experiments using sparks [15] or lasers [16] under similar ${\overset{∼}{L}}_{\mathrm{bbo}}$ conditions. During the subsequent positive pressure pulse period, the bubble–bubble interaction slightly advances the contraction time of bubble 2 and delays the contraction of bubble 1 compared to the single-bubble cases. However, the collapse times of bubbles appear close to those of the single-bubble cases. While the two bubbles rapidly contract and collapse, they attract each other, and then coalesce during the bubble re-expansion period.

The cell deformations associated with single and two-bubble expansion, contraction, collapse and re-expansion are depicted in Fig. 5b. Here, $D_{c}$ denotes the deformation of the top surface of the cell as defined in Fig. 1, and negative/positive signs represent compressive/expansive deformation. For the single-bubble cases, bubble 2, which is relatively far from the cell, has little effect on cell deformation during the whole period of bubble expansion and contraction, whereas bubble 1 causes cell deformation of $D_{c}=-1.09\;\mu \text{m}$ during the first period of bubble expansion. As bubble 1 contracts and collapses, the cell expands slightly, and then the re-expansion of bubble 1 causes a compressive cell deformation of $D_{c}=-0.76\;\mu \text{m}$. For the two-bubble case, the cell deformation is almost identical to that of the single-bubble case (bubble 1) before the collapse of bubble 1. Thereafter, a significant deformation of $D_{c}=-6.34\;\mu \text{m}$ occurs at the cell surface due to the interaction between collapsing bubbles, which will be described below.

**Fig. 6:**
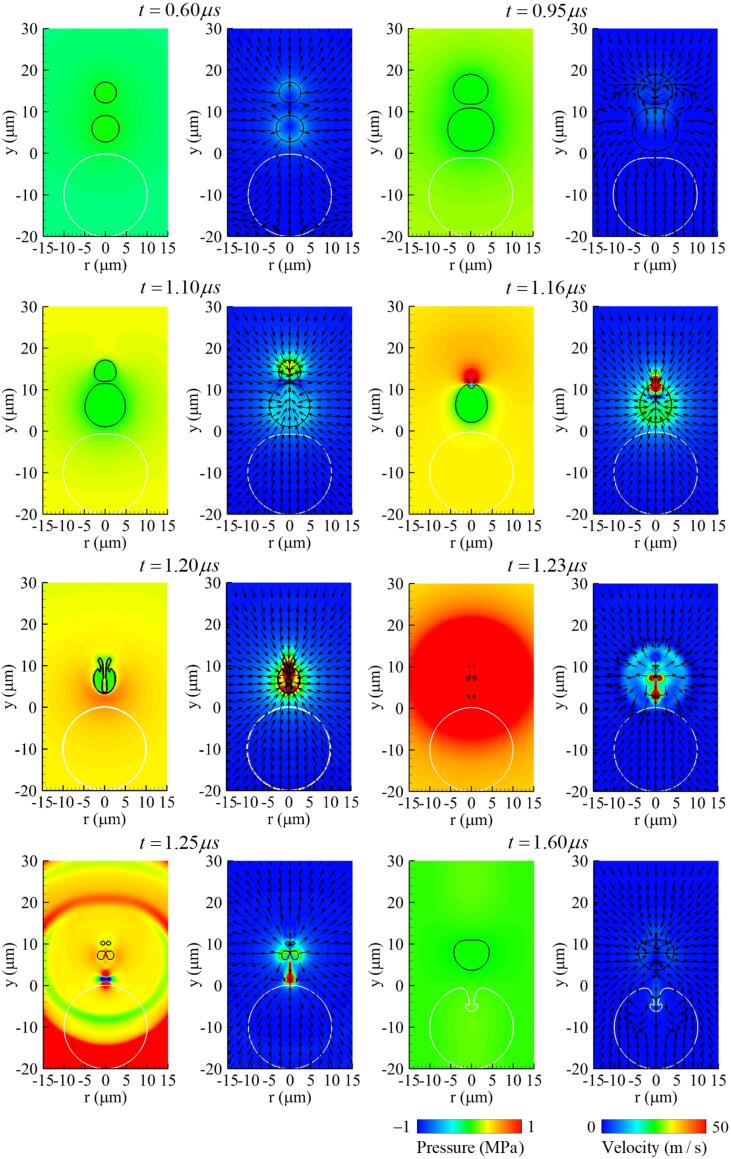
Instantaneous pressure field (left) and velocity field (right) associated with two-bubble oscillation near a deformable cell at $f=1\;\text{MHz},p_{A}=0.3\;\text{MPa},{\tilde{L}}_{\mathrm{bbo}}=0.8$ and ${\tilde{R}}_{\mathrm{bb},\max}=0.85$. Here, the black and white lines represent the bubble and cell surfaces, respectively.

**Fig. 7:**
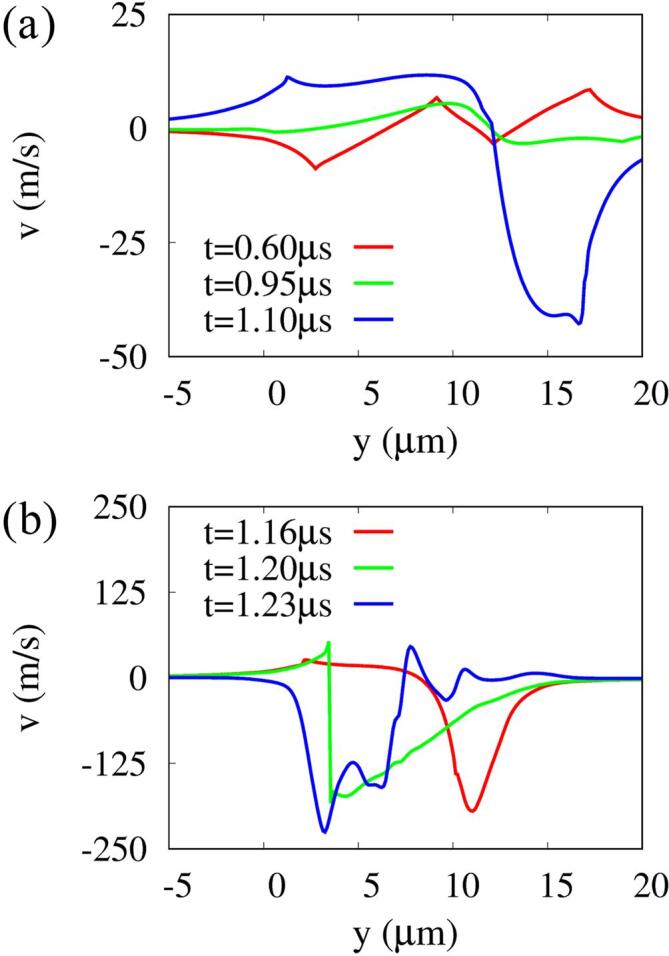
Velocity profiles along the y-axis at $f=1\;\text{MHz},p_{A}=0.3\;\text{MPa},{\tilde{L}}_{\mathrm{bbo}}=0.8$ and ${\tilde{R}}_{\mathrm{bb},\max}=0.85$ during (a) the bubble expansion and contraction period, and (b) the bubble collapse period.

Fig. 6, Fig. 7 present the pressure and velocity fields and the velocity profile along the y-axis associated with the two-bubble interaction and cell deformation. During the bubble expansion period of 0.6⩽t⩽0.95μs, the flows around the bubble surfaces adjacent to the two bubbles reduce each other’s expansion rates and the two surfaces become almost stationary, forming a thin liquid layer between the bubbles. As seen at t=1.10μs in Fig. 6, Fig. 7, during the contraction period of bubbles, attractive interactions occur between the bubbles and result in an upward flow of 10m/s around bubble 1 and a much faster downward flow of 40m/s around bubble 2, because bubble 1 has a larger negative force on bubble 2 as in the theoretical equation $p=(\rho_{l}/r)d(R_{b}^{2}{\overset{˙}{R}}_{b})/\mathrm{dt}$. The downward flow around bubble 2 is further increased by the mutual contraction of bubbles and becomes a fast liquid jet of 200m/s towards bubble 1 at t=1.16μs, when bubble 2 collapses. The liquid jet penetrates through bubble 1, as seen at t=1.20μs (Fig. 6, Fig. 7b). Thereafter, as bubble 1 collapses and expands again, a stronger shock wave is generated and propagated to the surroundings as depicted in Fig. S4 of the Supplementary information. The peak of the shock pressure decreases nearly proportional to $r^{-1}$ [5]. Strong pressure is applied to the upper surface of the cell, causing rapid deformation, but its influence on the cell deformations is not significant, as seen in Fig. 5c, because the period of the shock wave propagating at the sound speed to the cell is too short. On the other hand, the downward liquid jet generated by the collapse of bubble 2 increases at t=1.23μs to a velocity of 220m/s (Fig. 6, Fig. 7b) as bubble 1 collapses and expands again, and is transported to the upper surface of the cell. This causes a localized and deep compressive deformation at the cell surface, as seen at t=1.60μs in Fig. 6. This agrees well with the previous experimental observations [23], [24], [39] that cell perforation associated bubble collapse is caused by a liquid jet rather than a shock wave, and that the multi-bubble condition causes a stronger liquid jet than the single-bubble condition.

### 3.3 Influence of bubble–bubble distance

**Fig. 8:**
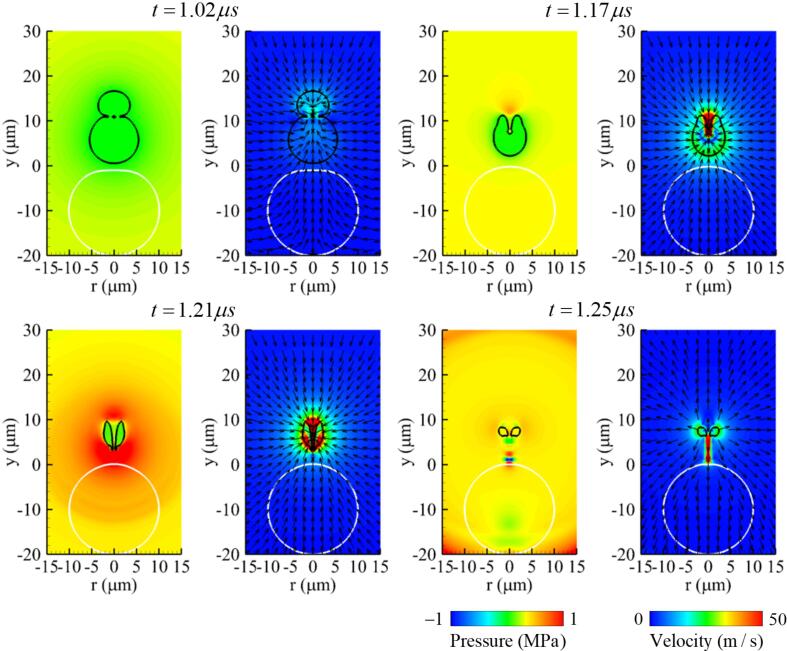
Instantaneous pressure field (left) and velocity field (right) associated with two-bubble oscillation near a deformable cell at $f=1\;\text{MHz},p_{A}=0.3\;\text{MPa},{\tilde{L}}_{\mathrm{bbo}}=0.6$ and ${\tilde{R}}_{\mathrm{bb},\max}=0.85$. Here, the black and white lines represent the bubble and cell surfaces, respectively.

Fig. 8 shows the results when the initial bubble–bubble distance is reduced to ${\overset{∼}{L}}_{\mathrm{bbo}}=0.6$ keeping other parameters the same as in the previous case. As the two bubbles expand and contract, they merge with each other at t=1.02μs under this bubble–bubble distance condition. This bubble coalescence condition is comparable with the experimental result of Chew et al. [15], where the spark-induced two bubbles were observed to merge with each other in the condition of ${\overset{∼}{L}}_{\mathrm{bbo}}<0.6$. The merged bubble is not spherical and has a greater curvature in its upper part than its lower part. The contraction proceeds rapidly in the upper part due to the greater positive pressure generated by the traveling ultrasound and its larger curvature, as described in Ref. [40]. This results in a liquid jet towards the bottom of the bubble at t=1.17μs. The liquid jet penetrates through the shrinking bubble at t=1.21μs and is then transported to the upper surface of the cell. The maximum jet velocity is about 145 m/s, which is lower than in the previous case of ${\overset{∼}{L}}_{\mathrm{bbo}}=0.8$.

**Fig. 9:**
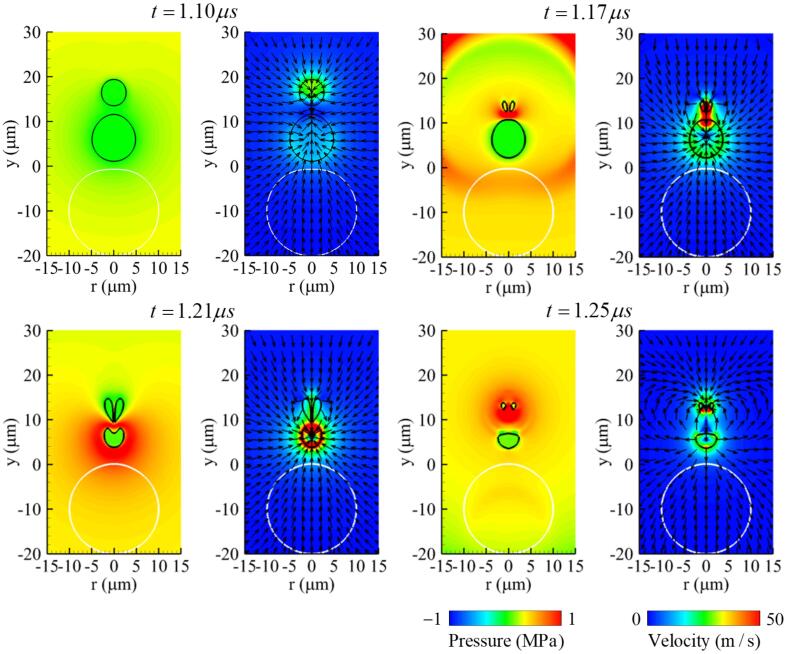
Instantaneous pressure field (left) and velocity field (right) associated with two-bubble oscillation near a deformable cell at $f=1\;\text{MHz},p_{A}=0.3\;\text{MPa},{\tilde{L}}_{\mathrm{bbo}}=1$ and ${\tilde{R}}_{\mathrm{bb},\max}=0.85$. Here, the black and white lines represent the bubble and cell surfaces, respectively.

The results at ${\overset{∼}{L}}_{\mathrm{bbo}}=1$ are plotted in Fig. 9. As the mutual interaction between bubbles decreases with increasing the initial bubble–bubble distance, the downward flow around bubble 2 is reduced to 30m/s at t=1.10μs. Thereafter, the collapse and re-expansion of bubble 2 generate a liquid jet towards bubble 1 at t=1.17μs. However, the liquid jet generated by bubble 2 is not strong enough to penetrate bubble 1, as seen at t=1.21μs, and disappears while bubble 1 collapses and expands again. The influence of the liquid jet on cell deformation is not observed at ${\overset{∼}{L}}_{\mathrm{bbo}}=1$, and this result is consistent with the empirical condition of ${\overset{∼}{L}}_{\mathrm{bbo}}<1$ observed in the experiment of Tomita and Sato [17] for the liquid jet to penetrate the bubbles in the two-bubble case.

**Fig. 10:**
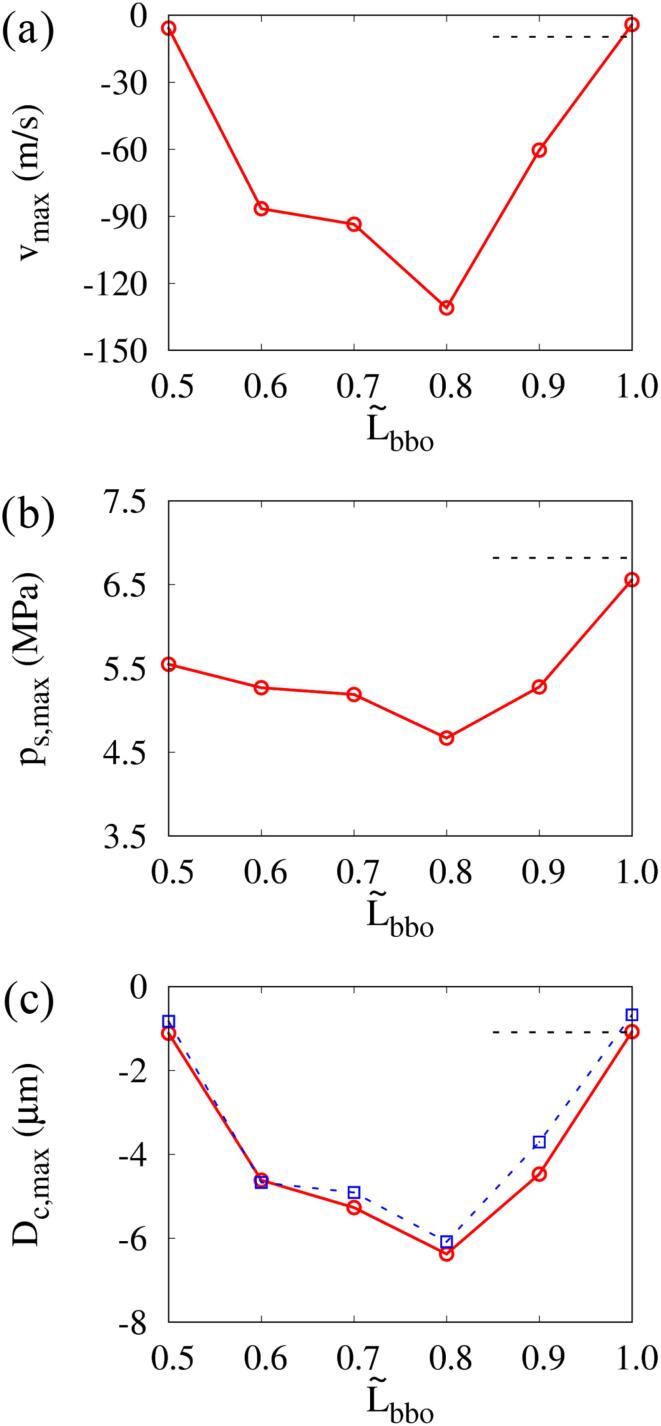
Effect of bubble–bubble distance on the shock wave pressure and jet-cell interaction at $f=1\;\text{MHz},p_{A}=0.3\;\text{MPa}$ and ${\tilde{R}}_{\mathrm{bb},\max}=0.85$: (a) maximum jet velocity at (r,y)=(0,0), (b) maximum pressure at $\left(r,y\right)=\left(15\;\mu \text{m},0\right)$ and (c) maximum cell deformation. The black dashed line represents the single-bubble case. The circle and square symbols represent the results of the two-bubble case and the predictions from the fitted equation using the results of $v_{\max}$, respectively.

Fig. 10 shows the effect of ${\overset{∼}{L}}_{\mathrm{bbo}}$ on the maximum liquid jet velocity $v_{\max}$ at (r,y)=(0,0) near the top surface of the cell, the maximum shock pressure $p_{s,\max}$ at (r,y)=(15μm,0) and the maximum cell deformation $D_{c,\max}$ while keeping ${\overset{∼}{R}}_{\mathrm{bb},\max}=0.85$. The results at ${\overset{∼}{L}}_{\mathrm{bbo}}=1$ are observed to be nearly similar to the single-bubble case. As ${\overset{∼}{L}}_{\mathrm{bbo}}$ decreases from 1, $\left|v_{\max}\right|$ increases until ${\overset{∼}{L}}_{\mathrm{bbo}}=0.8$ and then decreases whereas $p_{s,\max}$ is minimal at ${\overset{∼}{L}}_{\mathrm{bbo}}=0.8$, where $v_{\max}$ is the peak. This means that a large portion of the energy accumulated during bubble collapse is spent creating the liquid jet and less is distributed to the shock wave. Similarly, the experimental study of Cui et al. [41] on the interaction between the spark-induced two bubbles showed that the shock pressure was minimal at ${\overset{∼}{L}}_{\mathrm{bbo}}=0.8$ in the range of $0.5⩽{\overset{∼}{L}}_{\mathrm{bbo}}⩽1.1$. Under the condition of ${\overset{∼}{R}}_{\mathrm{bb},\max}=0.85$, as long as bubble 2 collapses before than bubble 1 with downward liquid jet, the jet direction does not change regardless of the change in ${\overset{∼}{L}}_{\mathrm{bbo}}$, which is consistent with the jet direction results of Refs. [15], [16]. As ${\overset{∼}{L}}_{\mathrm{bbo}}$ decreases below 1 and the attraction effect between the two bubbles increases, the maximum velocity $v_{\max}$ of the downward liquid jet caused by the collapse of bubble 2 becomes stronger and the maximum $D_{c,\max}$ of the resulting cell deformation increases. The curves of $v_{\max}$ and $D_{c,\max}$ have a peak at ${\overset{∼}{L}}_{\mathrm{bbo}}=0.8$, and decrease as ${\overset{∼}{L}}_{\mathrm{bbo}}$ further decreases and the two bubbles merge. The liquid jet and cell deformation are prominent in the range of $0.6⩽{\overset{∼}{L}}_{\mathrm{bbo}}⩽0.9$, where the two bubbles are close enough to attract each other but do not merge. The trends of $v_{\max}$ and $D_{c,\max}$ with respect to ${\overset{∼}{L}}_{\mathrm{bbo}}$ are similar and $D_{c,\max}$ is well fitted by $|D_{c,\max}|=0.27|v_{\max}{|}^{0.64}$, where $D_{c,\max}$ and $v_{\max}$ in μm and m/s, respectively.

### 3.4 Effect of bubble–bubble size ratio

**Fig. 11:**
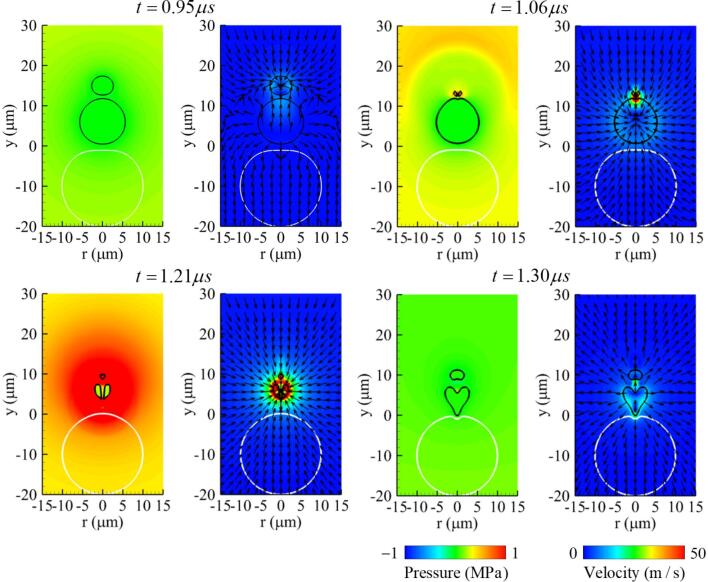
Instantaneous pressure field (left) and velocity field (right) associated with two-bubble oscillation near a deformable cell at $f=1\;\text{MHz},p_{A}=0.3\;\text{MPa},{\tilde{L}}_{\mathrm{bbo}}=0.8$ and ${\tilde{R}}_{\mathrm{bb},\max}=0.72$. Here, the black and white lines represent the bubble and cell surfaces, respectively.

Fig. 11 presents the results when the ratio of the radius of bubble 2 to bubble 1 is reduced to ${\overset{∼}{R}}_{\mathrm{bb},\max}=0.72$ (${\overset{∼}{R}}_{\mathrm{bbo}}=0.5$) while keeping ${\overset{∼}{L}}_{\mathrm{bbo}}=0.8$. At t=0.95μs near the maximum expansion of bubbles, the two bubbles do not merge, as in the previous case with ${\overset{∼}{R}}_{\mathrm{bb},\max}=0.85$ (Fig. 6). Thereafter, bubble 2 collapses forming a downward liquid jet of 160m/s at t=1.06μs, which is weaker and thinner than for ${\overset{∼}{R}}_{\mathrm{bb},\max}=0.85$ because the reduced expansion of bubble 2 results in a decrease in the contraction inertia. This matches well with the previous experimental observation [1] that the velocity and thickness of the liquid jet caused by bubble collapse increase proportionally to the maximum expansion of the bubble and its associated contraction inertia. The liquid jet reaches the top surface of the cell at t=1.21μs just before bubble 1 collapses, causing little deformation at the cell surface. On the other hand, as bubble 1 collapses and expands again, a liquid jet is ejected towards the cell at t=1.30μs. The resulting cell deformation is larger than that caused by the collapse of bubble 2 but less than for ${\overset{∼}{R}}_{\mathrm{bb},\max}=0.85$.

**Fig. 12:**
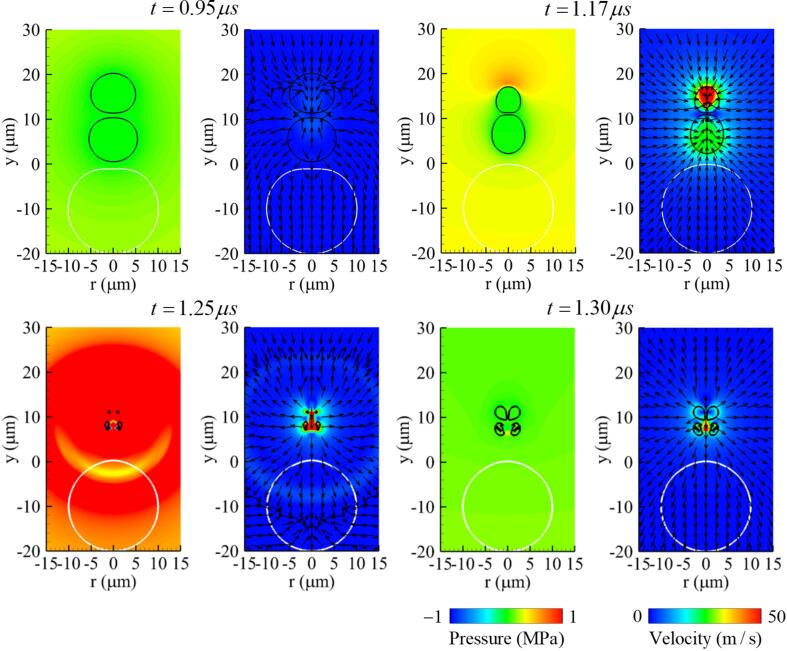
Instantaneous pressure field (left) and velocity field (right) associated with two-bubble oscillation near a deformable cell at $f=1\;\text{MHz},p_{A}=0.3\;\text{MPa},{\tilde{L}}_{\mathrm{bbo}}=0.8$ and ${\tilde{R}}_{\mathrm{bb},\max}=0.96$. Here, the black and white lines represent the bubble and cell surfaces, respectively.

The results at ${\overset{∼}{R}}_{\mathrm{bb},\max}=0.96$ (${\overset{∼}{R}}_{\mathrm{bbo}}=0.9$) are plotted in Fig. 12. As bubble 2 has a greater influence on bubble 1 with increasing size, the expansion of bubble 1 decreases, as seen at t=0.95μs. During the contraction period of bubbles, the interaction between bubbles causes an upward flow of 25m/s around bubble 1 and a downward flow 70m/s around bubble 2 at t=1.17μs, which are much higher than when bubble 2 has a similar size under the condition of ${\overset{∼}{R}}_{\mathrm{bb},\max}=0.85$, as seen at t=1.10μs in Fig. 6. The similar-sized two bubbles collapse with a short time difference near t=1.25μs, generating two individual liquid jets opposing each other. Thereafter, the two jets collide in the y=8μm plane, and the velocity decreases significantly to 60m/s toward the cell at t=1.30μs. The collided liquid jet travels downward but disappears before reaching the cell surface.

**Fig. 13:**
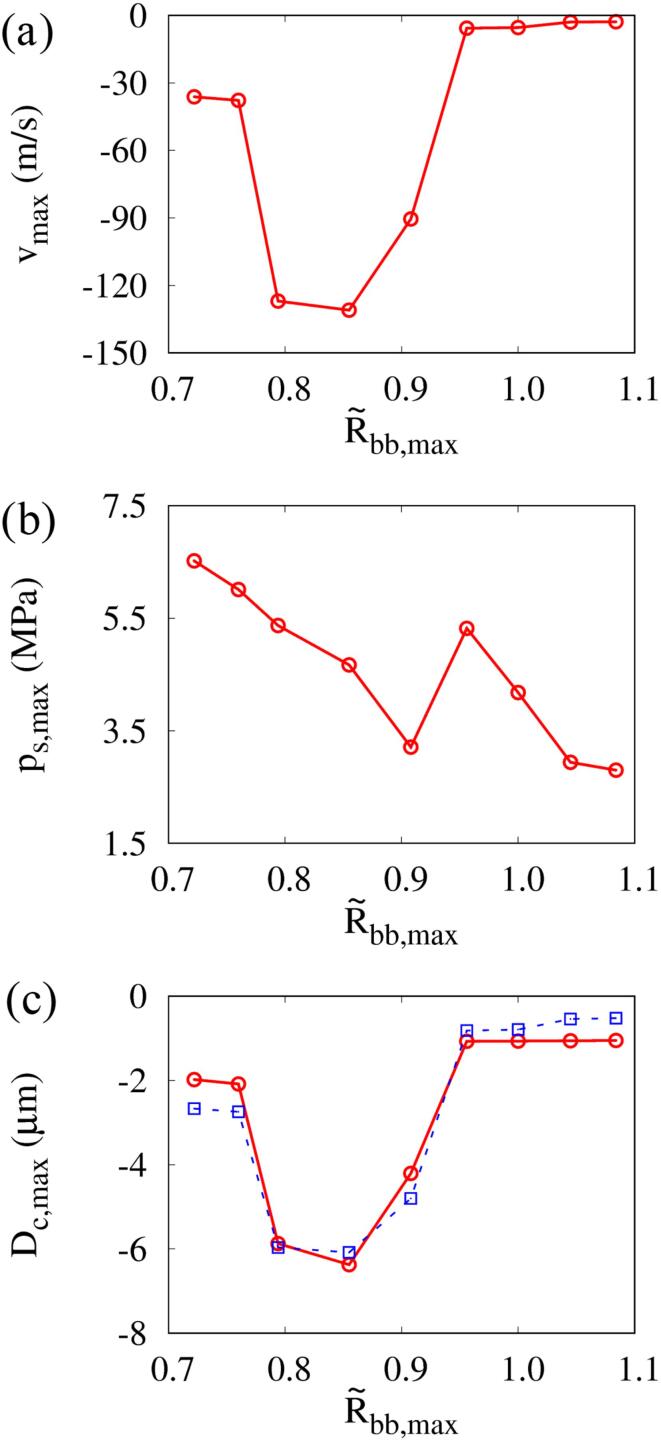
Influence of bubble–bubble size ratio on the shock wave pressure and jet-cell interaction at $f=1\;\text{MHz},p_{A}=0.3\;\text{MPa}$ and ${\overset{∼}{L}}_{\mathrm{bbo}}=0.8$: (a) maximum jet velocity at (r,y)=(0,0), (b) maximum pressure at (r,y)=(15μm,0) and (c) maximum cell deformation. The circle and square symbols represent the results of the two-bubble case and the predictions from the fitted equation using the results of $v_{\max}$, respectively.

Fig. 13 shows the influence of the bubble–bubble size ratio ${\overset{∼}{R}}_{\mathrm{bb},\max}$ on the maximum liquid jet velocity $v_{\max}$ at (r,y)=(0,0), the maximum shock pressure $p_{s,\max}$ at (r,y)=(15μm,0) and the maximum cell deformation $D_{c,\max}$ while keeping ${\overset{∼}{L}}_{\mathrm{bbo}}=0.8$. As ${\overset{∼}{R}}_{\mathrm{bb},\max}$ increases from 0.7, mutual repulsive and attractive interactions between the two bubbles increase, and $\left|v_{\max}\right|$ increases whereas $p_{s,\max}$ tends to decrease. However, in the range of $0.96⩽{\overset{∼}{R}}_{\mathrm{bb},\max}⩽1$, two opposite liquid jets collide with each other to form a water hammer shock, as observed in the experimental study of spark-induced two-bubble collapse [41]. This increases the shock pressure, but the maximum in that range is less than for a single bubble. As ${\overset{∼}{R}}_{\mathrm{bb},\max}$ increases from 0.7 and the expansion of bubble 2, which affects the upward and downward flows around the two bubbles, increases, $v_{\max}$ and $D_{c,\max}$ increase and have peaks at ${\overset{∼}{R}}_{\mathrm{bb},\max}=0.85$. The effect of the two-bubble interaction on $v_{\max}$ and $D_{c,\max}$ is pronounced in the range of $0.8⩽{\overset{∼}{R}}_{\mathrm{bb},\max}⩽0.9$. However, as ${\overset{∼}{R}}_{\mathrm{bb},\max}$ approaches 1, the two bubbles collapse almost simultaneously and the induced liquid jets collide with each other, reducing cell deformation. For ${\overset{∼}{R}}_{\mathrm{bb},\max}>1$, bubble 2 has a larger influence than bubble 1, bubble 1 collapses before bubble 2 with an upward liquid jet as depicted in Fig. S5 for ${\overset{∼}{R}}_{\mathrm{bb},\max}=1.08$ (${\overset{∼}{R}}_{\mathrm{bbo}}=1.2$) in the Supplementary information. $D_{c,\max}$ shows little difference in the range of ${\overset{∼}{R}}_{\mathrm{bb},\max}⩾1$. The equation of $|D_{c,\max}|=0.27|v_{\max}{|}^{0.64}$ used in the previous section also fits the relation between $v_{\max}$ and $D_{c,\max}$ well in a wide range of ${\overset{∼}{R}}_{\mathrm{bb},\max}$. Freund et al. [18] found that the deformed depth of tissue is proportional to the liquid jet velocity and inversely proportional to the viscosity of the tissue. Guo et al. [20] showed the maximum cell deformation increases as the liquid jet velocity increases depending on the acoustic frequency and amplitude. In their study, $v_{\max}=242\;\text{m}/\text{s}$ and $v_{\max}=388\;\text{m}/\text{s}$ result in $D_{c,\max}=0.98\;\mu \text{m}$ and $D_{c,\max}=1.37\;\mu \text{m}$, respectively, and this indicates that $\left|D_{c,\max}\right|$ is proportional to $|v_{\max}{|}^{0.71}$, which is comparable with the present fitting equation. Therefore, these observations mean that the liquid jet velocity is a key parameter that determines the cell deformation.

### 3.5 Influences of acoustic pressure amplitude and frequency

**Fig. 14:**
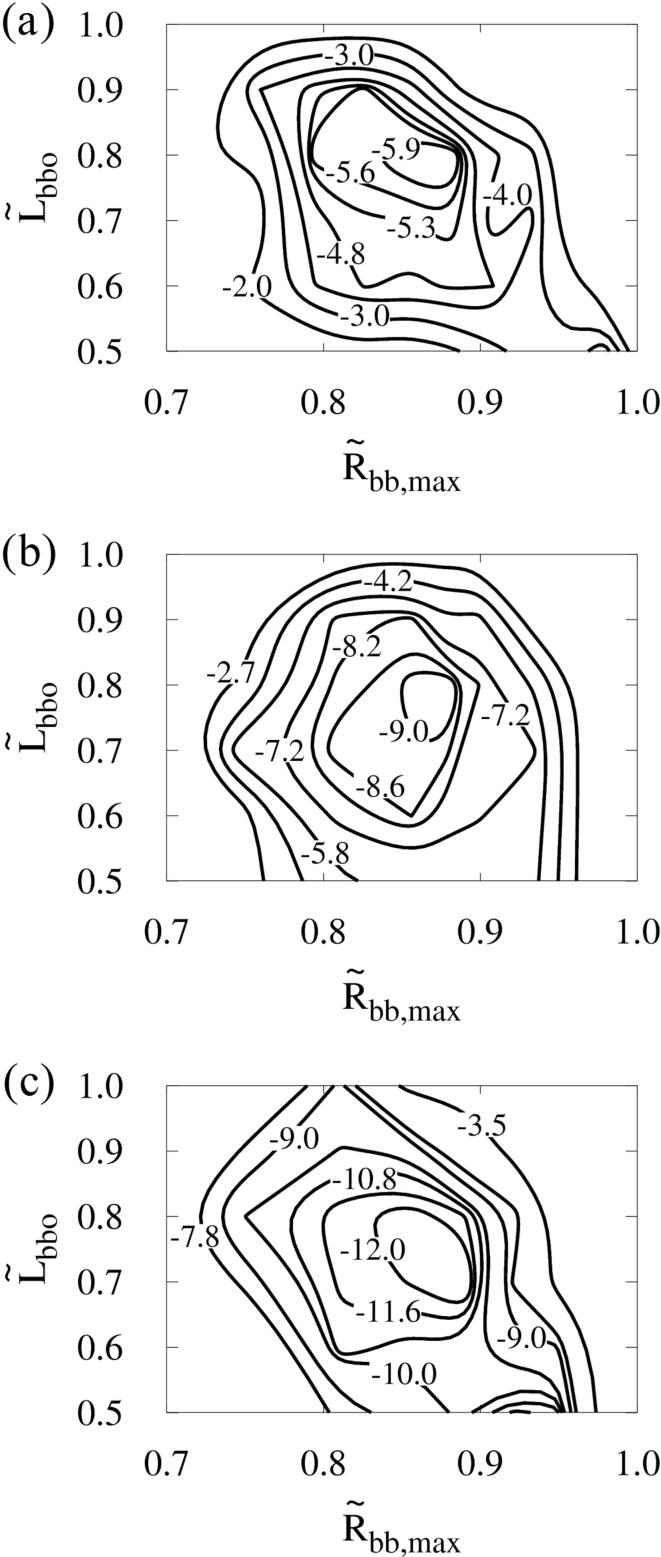
Combined effects of acoustic amplitude, bubble–bubble distance and size ratio on the cell deformation at f=1MHz: (a) $p_{A}=0.3\;\text{MPa}$, (b) $p_{A}=0.4\;\text{MPa}$ and (c) $p_{A}=0.5\;\text{MPa}$.

Fig. 14 presents the influence of the pressure amplitude $p_{A}$ of ultrasound on the maximum cell deformation $D_{c,\max}$ while maintaining the acoustic frequency at f=1MHz. The contour maps of $D_{c,\max}$ are obtained through extensive computations in the range of $0.5⩽{\overset{∼}{L}}_{\mathrm{bbo}}⩽1$ and $0.7<{\overset{∼}{R}}_{\mathrm{bb},\max}⩽1$. At $p_{A}=0.3\;\text{MPa}$, the optimal conditions for maximizing cell deformation are near ${\overset{∼}{L}}_{\mathrm{bbo}}=0.8$ and ${\overset{∼}{R}}_{\mathrm{bb},\max}=0.85$ (${\overset{∼}{R}}_{\mathrm{bbo}}=0.7$). $D_{c,\max}$ decreases sharply outside the narrow range of $0.7⩽{\overset{∼}{L}}_{\mathrm{bbo}}⩽0.9$ and $0.8⩽{\overset{∼}{R}}_{\mathrm{bb},\max}⩽0.9$ around the optimal point. As $p_{A}$ increases, the maximum cell deformation increases, but the optimal conditions for cell deformation are close to ${\overset{∼}{L}}_{\mathrm{bbo}}=0.8$ and ${\overset{∼}{R}}_{\mathrm{bb},\max}=0.85$ regardless of $p_{A}$. It is noted that the condition ${\overset{∼}{R}}_{\mathrm{bb},\max}=0.85$ corresponds to ${\overset{∼}{R}}_{\mathrm{bbo}}=0.7$ at $p_{A}=0.3\;\text{MPa}$ and ${\overset{∼}{R}}_{\mathrm{bbo}}=0.5$ at $p_{A}=0.5\;\text{MPa}$. This indicates that as $p_{A}$ increases, the difference between initial bubble sizes increases to maximize cell deformation. Compared with the results at $p_{A}=0.3\;\text{MPa}$, $D_{c,\max}$ at the optimal point increases by 52% at $p_{A}=0.4\;\text{MPa}$ and by 103% at $p_{A}=0.5\;\text{MPa}$, and thus varies as $p_{A}^{1.4}$.

**Fig. 15:**
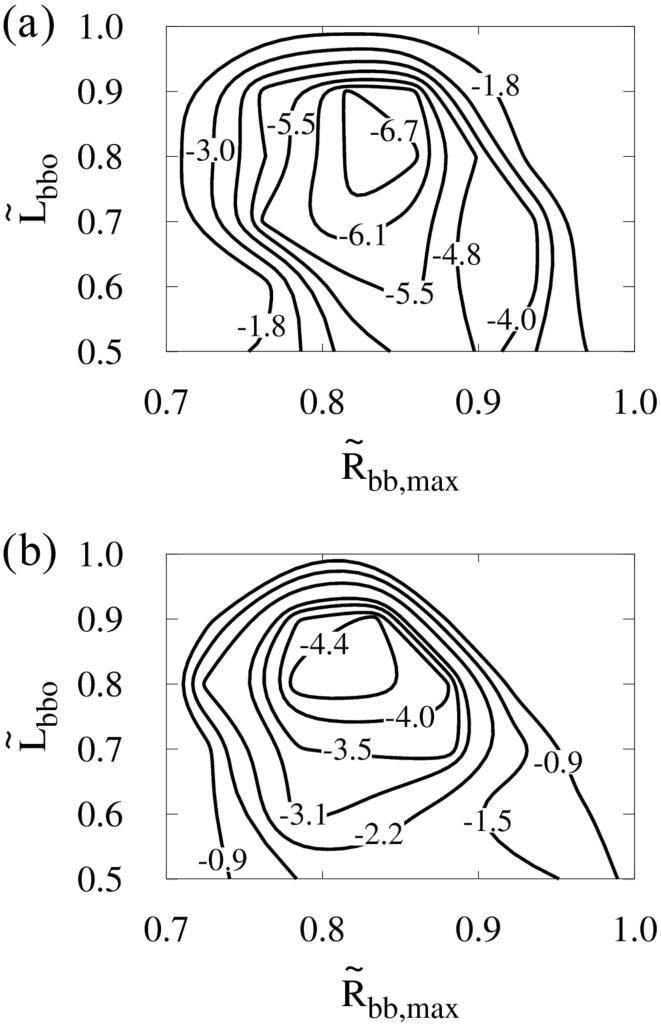
Combined effects of acoustic frequency, bubble–bubble distance and size ratio on the cell deformation at $p_{A}=0.5\;\text{MPa}$: (a) f=1.5MHz and (b) f=2MHz.

The effect of acoustic frequency *f* on $D_{c,\max}$ is plotted in Fig. 15 while keeping $p_{A}=0.5\;\text{MPa}$. Although *f* varies, the optimal conditions for $D_{c,\max}$ are consistently close to ${\overset{∼}{L}}_{\mathrm{bbo}}=0.8$ and ${\overset{∼}{R}}_{\mathrm{bb},\max}=0.85$. This indicates that the dimensionless parameters ${\overset{∼}{L}}_{\mathrm{bbo}}$ and ${\overset{∼}{R}}_{\mathrm{bb},\max}$ are the main determinants of the interactions between two bubbles and their influence on the deformation of the nearby cell. As *f* increases, the expansion of the bubbles decreases due to the reduced negative pulse period, and the collapse of the bubble becomes less violent [20]. Therefore, compared to the result at f=1MHz, $D_{c,\max}$ at the optimum point decreases by 45% at f=1.5MHz and 75% at f=2MHz, and thus varies as $f^{-1.44}$.

## 4 Conclusion

A numerical investigation of two-bubble interaction near a deformable cell in an ultrasonic field was presented by employing the level-set method for compressible multiphase flows with bubble and cell interfaces. The computations performed with different bubble–bubble distances and size ratios demonstrated a variety of bubble interactions, including bubble coalescence, bubble repulsion and attraction, jet penetration into the bubble, and jet collision. The interactions between the two collapsing bubbles formed a strong liquid jet and caused significant cell deformation compared to single-bubble collapse. The liquid jet and cell deformation were pronounced when the two bubbles were close enough to attract each other but did not merge. The maximum cell deformation was well fitted by a simple function of the maximum liquid jet velocity, indicating that the liquid jet velocity is a key parameter that determines the cell deformation. The contour maps of maximum cell deformation obtained through extensive computations showed that the optimal conditions for maximizing cell deformation are constant regardless of acoustic pressure amplitude and frequency, when using the dimensionless bubble–bubble distance and size ratio normalized to the maximum radius of a single bubble. This indicates that the dimensionless parameters are the main determinants of the interactions between two bubbles and their influence on the deformation of the nearby cell.

## CRediT authorship contribution statement

**Seongjin Hong:** Writing – original draft, Conceptualization, Software. **Gihun Son:** Writing – review & editing, Conceptualization, Methodology, Supervision.

## Declaration of Competing Interest

The authors declare that they have no known competing financial interests or personal relationships that could have appeared to influence the work reported in this paper.
