# Supplementary material for: Numerical investigation of two-microbubble collapse and cell deformation in an ultrasonic field
Source: Ultrason Sonochem. 2022 Dec 6;92:106252. doi: 10.1016/j.ultsonch.2022.106252 (PMC9730221; doi:10.1016/j.ultsonch.2022.106252)
Supplement: Supplementary data 1 [file mmc1.docx]

**Supplementary information**

**Numerical investigation of two-microbubble collapse and cell deformation in an ultrasonic field**

Seongjin Hong *^a^*, Gihun Son *^a,^* ^*^

*^a^* Department of Mechanical Engineering, Sogang University, 35 Baekbeom-ro, Mapo-gu, Seoul, 04107, South Korea

*^*^* E-mail address: [gihun@sogang.ac.kr](mailto:gihun@sogang.ac.kr) (Corresponding author: Gihun Son)


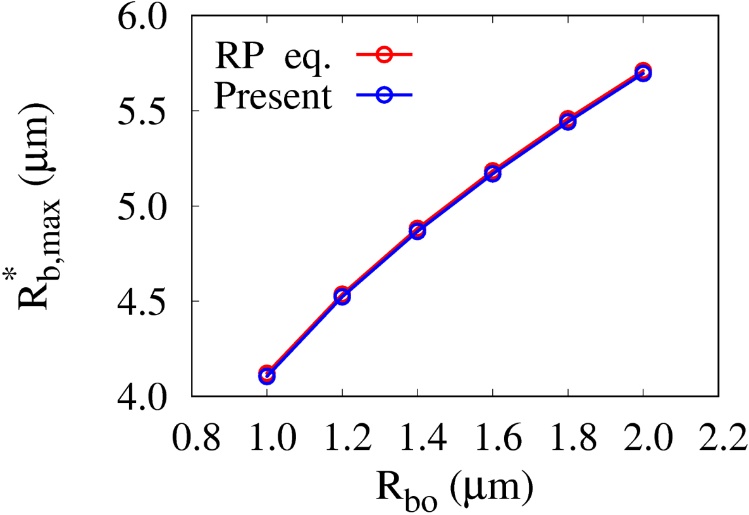


**Fig. S1** Maximum bubble radii for various initial bubble sizes at $f=1MHz$ and $p_{A}=0.3MPa$.


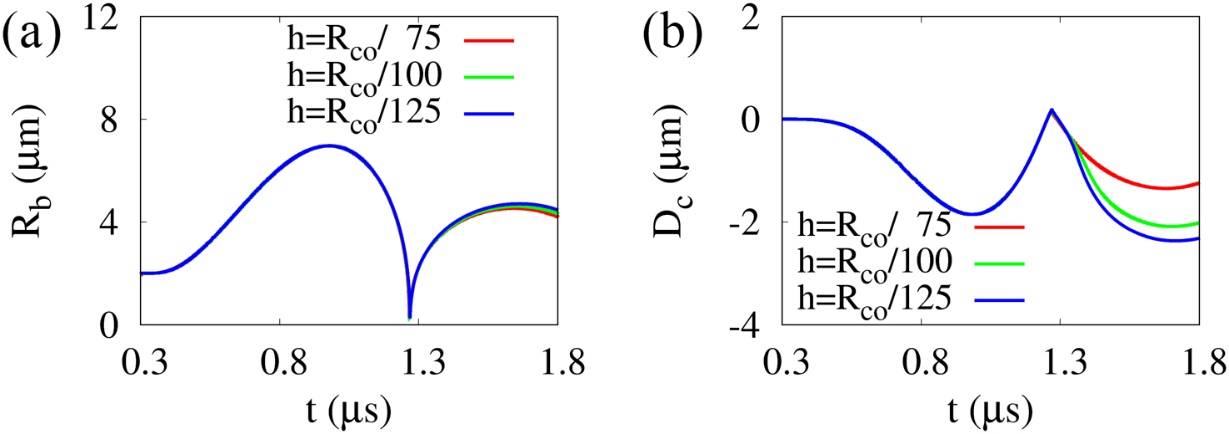


**Fig. S2** Grid convergence test on the ultrasound-induced oscillation of a single bubble near a deformable cell at $f=1MHz$, $p_{A}=0.4MPa$ and $N=1$: (a) bubble radius and (b) cell deformation.


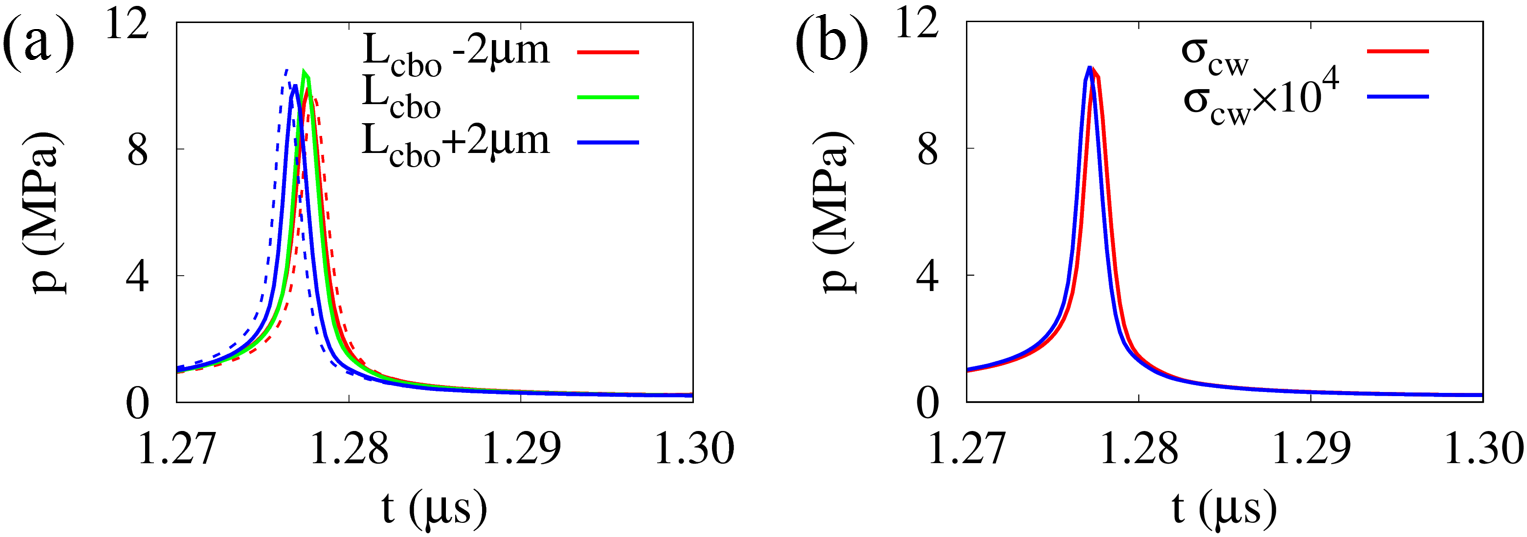


**Fig. S3** Temporal change of water pressure at $\left( r, y \right)=(15\mu m, 0)$ associated with a single microbubble near a deformable cell case at $f=1MHz$ and $p_{A}=0.4MPa$ depending on: (a) bubble-cell distance and (b) cell surface tension. The dash lines represent the results at $\left( r, y \right)=(15\mu m, \pm2\mu m)$ depending on the bubble-cell distances.


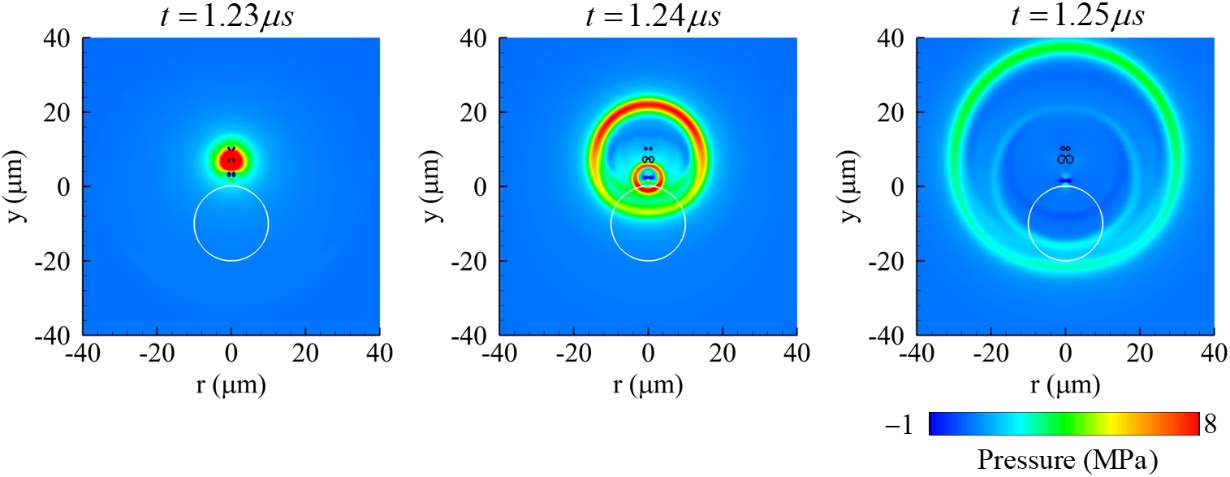


**Fig. S4** Instantaneous pressure field associated with shock waves caused by the collapse of the two-bubble near a deformable cell at $f=1MHz$, $p_{A}=0.3MPa$, $\tilde{L}_{bbo}=0.8$ and $\tilde{R}_{bb,max}=0.85$. Here, the black and white lines represent the bubble and cell surfaces, respectively.


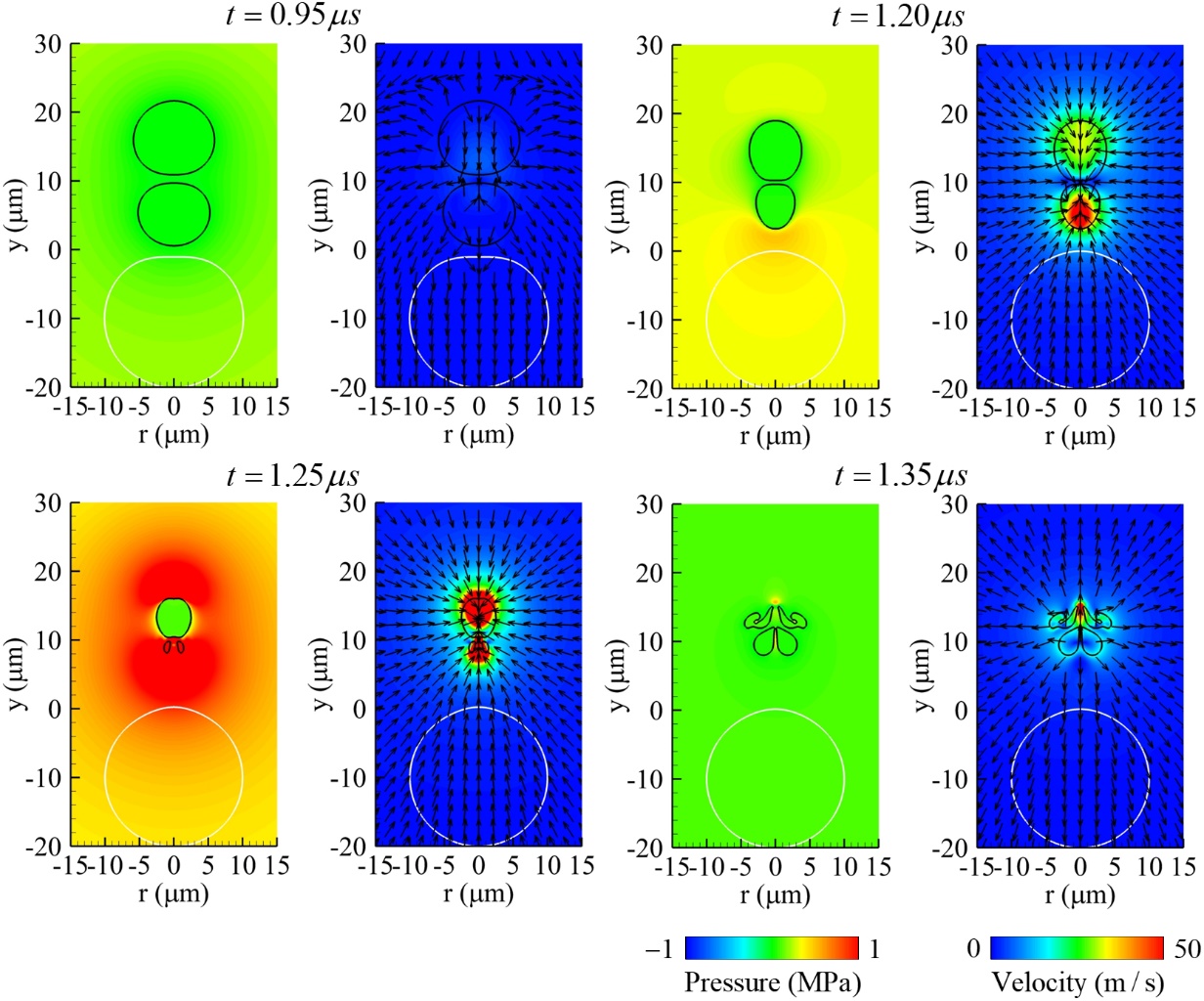


**Fig. S5** Instantaneous pressure field (left) and velocity field (right) associated with two-bubble oscillation near a deformable cell at $f=1MHz$, $p_{A}=0.3MPa$, $\tilde{L}_{bbo}=0.8$ and $\tilde{R}_{bb,max}=1.08$. Here, the black and white lines represent the bubble and cell surfaces, respectively.
